# Quantitation of Colonic Cells as Severity Markers in Patients with Irritable Bowel Syndrome

**DOI:** 10.22086/gmj.v0i0.1063

**Published:** 2018-05-26

**Authors:** Mohammad Bagher Miri, Amir Sadeghi, Afshin Moradi, Mohammad Rostami-Nejad, Hamid Asadzadeh Aghdaei, Mohammad Javad Ehsani Ardekani, Mohammad Taghi Safari, Mohammad Reza Zali

**Affiliations:** ^1^IBS Department, Gastroenterology and Liver Diseases Research Center, Research Institute for Gastroenterology and Liver Diseases, Shahid Beheshti University of Medical Sciences, Tehran, Iran; ^2^Department of Pathology, Shohada Hospital, Shahid Beheshti University of Medical Science, Tehran, Iran; ^3^Celiac disease Department, Basic and Molecular Epidemiology of Gastrointestinal Disorders Research Center, Research Institute for Gastroenterology and Liver Diseases, Shahid Beheshti University of Medical Sciences, Tehran, Iran

**Keywords:** Irritable Bowel Syndrome, Biopsy, Intra-epithelial Lymphocytes

## Abstract

**Background:**

Irritable bowel syndrome (IBS) is the most common gastrointestinal syndrome. Routine histopathology and immunohistochemistry (IHC) evaluations have shown an increase in the number of different inflammatory cells in the colon of IBS patients. In this study, we have compared the number of intraepithelial lymphocytes (IELs), eosinophils, mast cells and CD3+ T cells, in IBS patients and normal subjects.

**Materials and Methods:**

In 2016, seventynine patients with IBS and seventy-nine healthy subjects who underwent colonoscopy for other non-specific causes and with no pathologic findings, were enrolled in this cross-sectional study. Biopsy specimens obtained from the colon were stained, using IHC methods to determine the number of IELs, eosinophils, mast cells and CD3+ T cells. Quantitative and qualitative variables were compared between the two groups, using a Chi-square test and Student’s t-test.

**Results:**

Seventy-nine patients with IBS, 79.7% females with a mean age of 42.5±14.6 years, were recruited, as the case group, and seventy-nine individuals, 51.9% females with a mean age of 39.7±18.9 years, were enrolled as controls. The average number of IELs per high power fields (hpf) was found to be higher in the IBS group, and this difference was statistically significant (32.8±11.8 vs. 28.6±12.9; P=0.034). Also, the mean count/hpf of CD3+ T lymphocytes (23.1±7.9 vs. 20.2±8.1; P=0.024) and mast cells (7.6±3.1 vs. 6.6±3.0; P=0.041) were significantly higher in the IBS group, compared to the control group. The number of eosinophils was higher in the IBS group, but the differences were not statistically significant (P=0.066).

**Conclusion:**

According to the results, we suggest that analysis of immune cells and IELs in intestinal biopsies might be an appropriate method for diagnosis of IBS.

## Introduction

Irritable bowel syndrome (IBS) is the most common gastrointestinal syndrome with a prevalence of 10-15 percent in the general population, with a peak in the third and fourth decades of life. The risk of developing IBS in women is twice the risk, calculated for men [1].

This disease usually presents with intermittent diarrhea and constipation. Unlike ulcerative colitis and Crohn’s disease, IBS is not associated with significant histological changes in the intestines, and it is not pre-cancerous. Various drugs have been developed for the treatment of IBS; however, due to the lack of knowledge about its pathophysiology, there is still no definite cure for this disease [2].

Immunologic factors have been proposed to be involved in the pathogenesis of IBS. However, the relative effect of this immune component and its correlation with gender and gastrointestinal complaints in IBS patients’ remains poorly understood. Current evidence suggests a reduced secretion of chemokines (IL-8, CXCL-9, and MCP-1) rather than pro-inflammatory cytokines (TNF-alpha, IL-6 and IL-1beta) [3]. Proteinase-activated receptor 2 (PAR-2) has also been shown to be involved in the pathogenesis of IBS when activated by tryptase from mast cells or luminal proteases [4]. Elevated colonic luminal serine protease activity in IBS-D (IBS with diarrhea) patients, evokes a PAR-2-mediated colonic epithelial barrier dysfunction, suggesting a role in the pathogenesis of IBS [5].

Recent studies have shown an increased number of IELs, mast cells and enterochromaffin cells (EC), in immunohistochemical (IHC) assessments of the colon tissue. Female patients were found to have a higher number of mast cells, compared to males, associated with the frequency of abdominal bloating and symptoms of dysmotility-like dyspepsia [6]. Furthermore, a role of the mucosal immune system in the pathogenesis of IBS is suggested, by its association with intestinal infections [7]. Those who develop IBS after gastroenteritis shows an increased number of ECs and lymphocyte cell count, compared with those who do not develop IBS. The alterations in the number of ECs, mast cells, and lamina propria T lymphocytes, in IBS patients have also been shown to be related to psychological factors [8]. Accordingly, we aimed to compare the number of IEL, eosinophils, mast cells and CD3+ T cells in IBS patients with normal subjects, in order to identify the appropriate diagnostic markers for IBS diagnosis.

## Materials and Methods

### Study Design and Sample Population

In this cross-sectional study, seventy-nine patients diagnosed with IBS in 2016, based on the ROME IV criteria [9], referred to the gastroenterology clinic of the Shohadaye Tajrish Hospital, Tehran, Iran, were included as the case group. According to their predominant symptoms, patients were classified as IBS-C (constipation), IBS-D (diarrhea), IBS-M (mixed) and IBS-U (unclassified).

### Data Collection

Demographic characteristics of the patients, along with their medical history, including their symptom specifications were recorded by a Gastroenterologist, using a questionnaire. Data regarding the histopathological evaluations, including the count of IELs, eosinophils, mast cells and CD3+ T lymphocytes were later added to the datasheets.

Hence, the inclusion criteria for the case group were as follows: having recurrent abdominal pain at least one day per week, in the past three months. Patients were excluded from the study if they had other gastrointestinal symptoms, not resembling IBS or suggestive of a diagnoses other than IBS such as celiac disease, or those with abnormal findings in their colonoscopy.

All subjects underwent a colonoscopy, and after confirming the normal colonic appearance, four biopsy specimens were obtained from different parts of their colon and stored in formalin containers, for further histopathological evaluations.

Seventy-nine subjects, without irritable bowel symptoms, were also included as the control group. These subjects were recruited, through convenience sampling methods, from those individuals who underwent colonoscopy for reasons other than evaluation of upper or lower gastrointestinal symptoms, and with no significant abnormalities in their colon. However, those with constipation, evidence of rectal mucosal erythema or mucosal prolapse, history of medications, particularly corticosteroids and anti-inflammatory agents were excluded. Similar to the case group, four biopsy specimens were obtained from different parts of the colon and sent for histopathological evaluations.

### IHC Protocol

Biopsy specimens were fixed in formalin, and paraffin-embedded tissue sections were cut at 10 mm×3 mm×10 mm. Freshly dissected tissues (thickness <4mm) were fixed with 2% paraformaldehyde for a mean duration of 3 hours at room temperature, and then rinsed with running tap water for 5 min. Then the tissue was dehydrated, by applying 70%, 80%, 95% and eventually three times of 100% alcohol, each for 5 min. Samples were then exposed to butanol for 24 hours. The tissue samples were washed in xylene twice, and then immersed in paraffin three times, each for 5 min. The tissues were then embedded in a 56-60°C paraffin block for 2 hours and refrigerated. The paraffin-embedded tissue blocks were sectioned on a microtome at 5-8 μm thickness and then suspended in 40°C water bath, containing distilled water. Sections were then transferred onto glass slides suitable for immunohistochemistry, and dried overnight at room temperature. Slides were deparaffinized in xylene twice, transferred into 100% alcohol twice, and then transferred once through 95%, 70% and 50% alcohols, for 5, 3 and 3 min each, respectively. Endogenous peroxidase activity was blocked by incubating sections, in 3% H_2_O_2_ solution in methanol at room temperature for 10 min. Slides were rinsed with Phosphate Buffered Saline (PBS) twice, 100 μL blocking buffer (e.g., 10% fetal bovine serum in PBS) was added onto the sections of the slides, and incubated in a humidified chamber at room temperature for one hour. Then 100 μL appropriately diluted primary antibody (in antibody dilution buffer, e.g., 0.5% bovine serum albumin in PBS) was applied to the sections on the slides, and incubated in a humidified chamber at room temperature for one hour. Then 100 μL appropriately diluted biotinylated secondary antibody (using the antibody dilution buffer) was applied to the slides, and similarly incubated for 30 min. Then 100 μL diluted Sav-HRP conjugates (using the antibody dilution buffer) were applied to the sections and incubated for 30 min (kept protected from light). Subsequently, 100 μL DAB substrate solution (freshly made just before use: 0.05% DAB − 0.015% H2O2 in PBS) was applied to the slides to reveal antibody staining. In less than 5 min, the desired color intensity was reached. The slides were rinsed in running tap water for 10 min and dehydrated through cycles of alcohol application (95%, 95%, 100% and 100%), each cycle for 5 min. The tissue slides were washed three times in xylene and coverslip, using mounting solution, and then evaluated under microscope.

All the prepared slides were assessed by an expert Pathologist, who was blinded to the diagnosis of the patients. To determine the number of IELs, eosinophils, mast cells and CD3+ T lymphocytes, five non-overlapping hpf, with a magnification of 400× were evaluated, and the average of these five fields was reported as the count/hpf.

### Ethical Consideration

The study protocol was reviewed and approved by The Ethical Committee of Research Institute for Gastroenterology and Liver Diseases, Shahid Beheshti University of Medical Sciences (IR.SBMU.RIGLD.1395.35) All the procedures were in accordance with the ethical standards of the institutional research committee, and the guidelines of Helsinki’s Declaration. Information about the study was given comprehensively, both orally and in written form, to all patients or accompanying guardian. An informed written consent was obtained from all participants, prior to participation in the study.

### Statistical Analysis

SPSS software, version 20.0 (SPSS Inc., Chicago, IL, USA) was used to perform all statistical analyses, in this study. Quantitative and qualitative variables were respectively presented, as mean±standard deviation (SD) and frequency (percentage). Qualitative and quantitative variables were compared between the two groups, using a Chi-square test and Student’s t-test, respectively. A p value of less than 0.05 was considered statistically significant.

## Results

Seventy-nine patients with IBS, 79.7% females with a mean age of 42.5±14.6 years, were recruited as cases, and seventy-nine individuals, 51.9% females with a mean age of 39.7±18.9 years, served as controls. The gender difference between the two groups was statistically significant (P<0.001). The mean age of patients in the IBS group was not significantly higher than the control group (P=0.3). The majority of patients had diarrhea-predominant IBS (59.5%), while the predominant symptom in 15 subjects (19%) was constipation, nine (11.4%) had mixed IBS, and eight subjects (10.1%) could not be classified in any of these groups.

**Figure-1:**
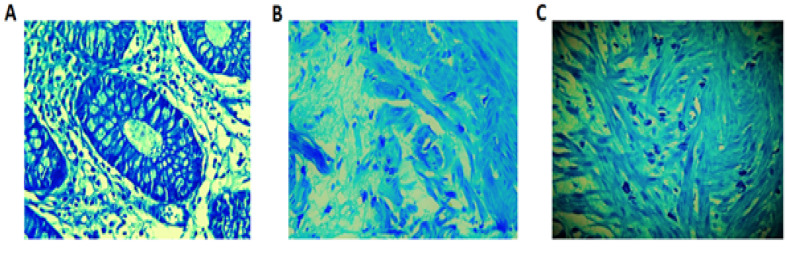
Comparison between tryptase-positive mast cells in the colonic mucosa of a healthy control subject and IBs patients by photomicrographs. Normal control **(A)**: hpf of colonic mucosa with no mast cells, Giemsa staining. IBS case **(B)**: Colonic mucosa with an increased number of mast cells, some of them have ruptured membrane, Giemsa staining. IBS case **(C)**: Colonic muscularis mucosa with an increased number of mast cells, Giemsa staining, the original magnification of 400x.

As presented in Table-1, the mean number of IELs per hpf was higher in the IBS group, and the difference was statistically significant (P=0.034). Similarly, the mean count/hpf of CD3+ T lymphocytes (P=0.024) and mast cells (P=0.041) were also significantly higher in the IBS group, compared to the control group. However, the number of eosinophils was higher in the IBS group, but the difference was not statistically significant (P=0.066). Comparison between tryptase-positive mast cells in the colonic mucosa of healthy control subjects and IBD patients by photomicrographs are presented in Figure-1.

**Table-1:** Demographic Characteristics and Immune Cell Count in the Two Groups of IBS and Control

**Variables**	**IBS (n=79)**	**Control (n=79)**	**P-value**
**Age** (mean±SD)	42.5±14.6	39.7±18.9	0.3
**Sex** Male Female	16 (20.3%) 63 (79.7%)	38 (48.1%) 41 (51.9%)	<0.001
**Predominant symptom** Diarrhea Constipation Mixed Unclassified	47 (59.5%) 15 (19.0%) 9 (11.4%) 8 (10.1%)	- - - -	NA
**Immune cell count/hpf**			
Intra-epithelial lymphocyte	32.8 ± 11.8	28.6 ± 12.9	0.034
CD3+ T lymphocyte	23.1 ± 7.9	20.2 ± 8.1	0.024
Eosinophil	5.4 ± 2.9	4.5 ± 3.2	0.066
Mast cell	7.6 ± 3.1	6.6 ± 3.0	0.041

**NA:** Not Applicable

## Discussion

The present study investigated the subtle histopathological changes in the colon of patients, diagnosed with IBS by comparing the number of IELs, CD3+ T cells, eosinophils and mast cells in the colon biopsies with normal subjects. The results of this study showed that the numbers of all evaluated cell types were higher in the IBS patients, and only the difference in the number of eosinophils was not statistically significant. Overall, our findings were indicative of an increase in immune system activation in IBS patients.

IBS is a common functional disorder without a defined pathology. Numerous studies have tried to identify the contributing factors in the development of this disease, some of which have suggested that inflammation and neuronal degeneration in the myenteric plexus, might be involved in the pathogenesis of IBS [9]. In this regard, attention has been drawn to the cellular and molecular aspects of IBS, particularly the changes in the numbers of lymphocytes, eosinophils, mast cells, and other inflammatory cells in the colonic mucosa of IBS patients.

Several studies have confirmed increased numbers of T lymphocytes, both in the lamina propria [9, 10] and epithelium of the colonic crypts in IBS patients [11, 12], with greater increases in diarrhea-predominant IBS, rather than the constipation-predominant form [13]. In one of the earlier studies, Gwee *et al*., evaluated 94 patients with gastroenteritis and reported that three months after the infection, rectal biopsy specimens from IBS patients, showed an increased chronic inflammatory cell counts, while specimens obtained from other subjects had returned to normal levels [14]. They also found that increases in the number of IELs were associated with simultaneous increases in the number of CD3+ T lymphocytes. Lee *et al*., also showed that in patients, diagnosed with IBS after a *Campylobacter* infection, the number of rectal mucosal ECs, CD3+, CD4+, and CD8+ lymphocytes in the lamina propria were significantly higher than that of healthy controls [15].

Dunlop *et al*., aimed to investigate whether histological or clinical features of post-infective IBS [PI-IBS] are different from those without a history of infection. They found increases in the number of lamina propria T lymphocytes, in both PI-IBS and non–PI-IBS patients, compared to healthy controls [16]. Hence, the findings of all these studies were consistent with our results, indicating an increase in the number of both IELs and CD3+ T-lymphocytes in the colonic mucosa of patients with IBS.

As for the eosinophils, these cells have been shown to be typically associated with allergic reactions, and studies have reported no significant changes in the level of eosinophil cationic protein or in their numbers, neither in blood nor in intestinal biopsies of patients with IBS [8]. Therefore, considering the differences in the number of eosinophils between IBS patients and healthy subjects, our results were similar to what has been shown in previous studies.

Some studies conducted in patients with diarrhea-predominant IBS have reported an increased number of mast cells in the cecum, terminal ileum, and jejunum of these patients [6, 17, 18]. Ford *et al*., suggested that low-grade mucosal inflammation, particularly mast cell activation, might be contributing to the pathogenesis of IBS and they emphasized on the importance of further assessments of mast cell stabilizers, as a potential treatment for IBS patients [9]. However, in an earlier study conducted by Hahn *et al*. , no significant increase in the number of mast cells was observed in the terminal ileum and colon of IBS patients [19]. Hence, compatible with most of these studies, we found a significant increase in the number of mast cells in IBS patients, compared to the control group. These immune cells release various sensitizing mediators, such as prostaglandins, substance P, and nerve growth factor, which can in turn provide a peripheral mechanism of sensitization of spinal nociceptive pathways, and their increased numbers in IBS patients may contribute to visceral hypersensitivity, shown to be correlated with abdominal pain and altered colonic motility [20, 21]. In this regard, Faure *et al*., have indicated that serotonin and 5-HT can independently promote eosinophils chemotaxis, and induce adhesion and migration of mast cells and eosinophils, that eventually lead to inflammation, involved in the development of IBS [22]. In addition, major basic protein released from eosinophils can also induce dysfunction of M2 receptors in the vagus nerve, resulting in increased GI motility. Most studies evaluating the changes in the number of immune cells in the colonic biopsies of IBS patients have confirmed increases in the numbers of IELs, CD3+ T lymphocytes, mast cells and even eosinophils, and have provided valuable evidence for an immune pathology in IBS. However, application of these findings in the diagnosis of IBS seems impractical considering the extent of overlap between affected and normal subjects, the invasive process of obtaining colonic biopsies, and the time-consuming quantitative histopathological assessments. However, these findings may pave the way for the investigations of therapeutic methods that counteract the proposed inflammation and immune activation pathways in IBS patients. These results could also be utilized for finding potential diagnostic markers for IBS, based on the substances that these specific immune cells release in the body. One of the limitations of this study was the small sample population. Moreover, evaluation of the occurrence of IBS symptoms post-gastrointestinal infection relied on subject recall and was thus prone to error.

## Conclusion

The findings of this study showed greater numbers of IELs, CD3+ T lymphocytes, mast cells, and eosinophils in the colonic biopsies of patients with IBS, compared to the healthy controls, which is indicative of inflammation and immune activation in the colon of IBS patients. These findings could pave the way for further investigations of IBS treatments and diagnostic markers.

## Conflict of Interest

The authors declare no conflict of interest.
